# Tetra­ammine-2κ^4^ *C*-μ-cyanido-1:2κ^2^ *C*:*N*-tricyanido-1κ^3^ *C*-copper(II)palladium(II)

**DOI:** 10.1107/S1600536813011033

**Published:** 2013-04-30

**Authors:** Veronika Suchá, Juraj Kuchár, Klaus Harms

**Affiliations:** a Department of Inorganic Chemistry, Institute of Chemistry, P.J. Šafárik University in Košice, Moyzesova 11, 041 54 Košice, Slovakia; b Fachbereich Chemie der Philipps Universität, Hans-Meerwein-Strasse, D-35043 Marburg, Germany

## Abstract

The title compound, [Cu(NH_3_)_4_-(μ_2_-NC)—Pd(CN)_3_], is a binuclear copper(II)palladium(II) complex, in which the Cu^II^ coordination is defined by four ammine ligands and one bridging cyanide ligand. The Cu—N bond lengths in the base of the resulting CuN_5_ pyramid are in the range 2.016 (3)–2.024 (3) Å and the apical Cu—N( C) distance is 2.385 (4) Å. Based on the τ parameter, the shape of the coordination polyhedron is tetra­gonal–pyramidal (τ = 0). All atoms of the square-planar tetracyanidopalladate(II) moiety and the Cu^II^ ion are located on a mirror plane. The electroneutral mol­ecules inter­act by N—H⋯N hydrogen bonds, resulting in the formation of a three-dimensional network.

## Related literature

For related crystal structures of Cu^II^ complexes see: Escorihuela *et al.* (2001 ▶); Seitz *et al.* (2001 ▶); Kuchár *et al.* (2004 ▶). For additional analysis of structural parameters, see: Addison *et al.* (1984 ▶).
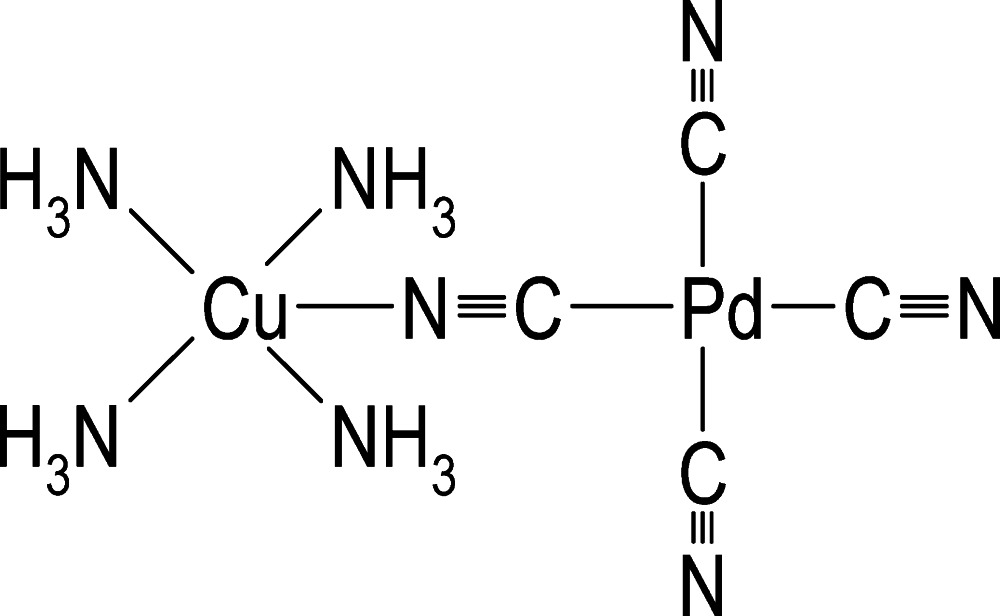


## Experimental

### 

#### Crystal data

[CuPd(CN)_4_(NH_3_)_4_]
*M* *_r_* = 342.17
Orthorhombic, 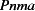

*a* = 14.5204 (9) Å
*b* = 7.2358 (5) Å
*c* = 10.3955 (6) Å
*V* = 1092.22 (12) Å^3^
*Z* = 4
Mo *K*α radiation
μ = 3.57 mm^−1^
*T* = 100 K
0.3 × 0.1 × 0.1 mm

#### Data collection

Stoe IPDS-II diffractometer
Absorption correction: multi-scan (Blessing, 1995 ▶) *T*_min_ = 0.332, *T*_max_ = 0.646
4036 measured reflections
1051 independent reflections
958 reflections with *I* > 2σ(*I*)
*R*_int_ = 0.032

#### Refinement

*R*[*F* ^2^ > 2σ(*F* ^2^)] = 0.025
*wR*(*F* ^2^) = 0.061
*S* = 1.00
1051 reflections
81 parameters
H-atom parameters constrained
Δρ_max_ = 0.54 e Å^−3^
Δρ_min_ = −1.26 e Å^−3^

### 

Data collection: *X-AREA* (Stoe & Cie, 2002 ▶); cell refinement: *X-AREA*; data reduction: *X-AREA*; program(s) used to solve structure: *SHELXS97* (Sheldrick, 2008 ▶); program(s) used to refine structure: *SHELXL97* (Sheldrick, 2008 ▶); molecular graphics: *DIAMOND* (Crystal Impact, 2009 ▶); software used to prepare material for publication: *SHELXL97*.

## Supplementary Material

Crystal structure: contains datablock(s) I, global. DOI: 10.1107/S1600536813011033/ff2104sup1.cif

Structure factors: contains datablock(s) I. DOI: 10.1107/S1600536813011033/ff2104Isup2.hkl

Additional supplementary materials:  crystallographic information; 3D view; checkCIF report

## Figures and Tables

**Table 1:** Selected bond lengths (Å)

Pd1—C4	1.985 (5)
Pd1—C2	1.992 (5)
Pd1—C3	1.994 (5)
Pd1—C1	2.005 (5)
Cu1—N6	2.016 (3)
Cu1—N5	2.024 (3)
Cu1—N4	2.385 (4)

**Table 2:** Hydrogen-bond geometry (Å, °)

*D*—H⋯*A*	*D*—H	H⋯*A*	*D*⋯*A*	*D*—H⋯*A*
N5—H5*A*⋯N1	0.91	2.34	3.237 (4)	167
N5—H5*C*⋯N3^i^	0.91	2.39	3.267 (4)	162
N5—H5*B*⋯N3^ii^	0.91	2.65	3.180 (4)	118
N5—H5*B*⋯N4^iii^	0.91	2.52	3.297 (4)	144
N6—H6*B*⋯N1^ii^	0.91	2.53	3.131 (4)	124
N6—H6*B*⋯N2^iii^	0.91	2.58	3.348 (4)	142
N6—H6*A*⋯N3^i^	0.91	2.31	3.199 (4)	165

Symmetry codes: (i) 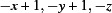; (ii) 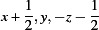; (iii) 
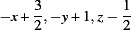
.

**Fig. 1.**
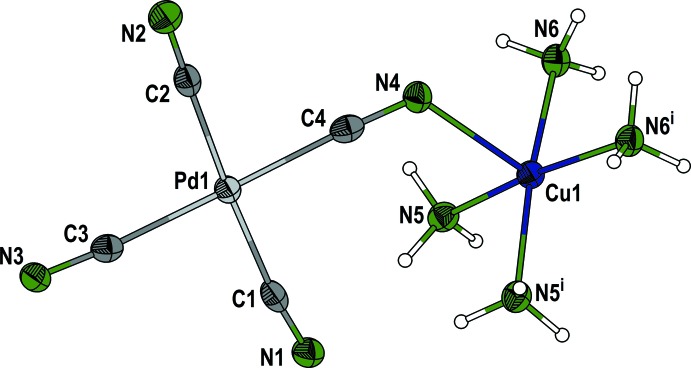
Molecular structure of the title molecule 1 with the atom-numbering scheme. Displacement ellipsoids are drawn at the 50% probability level. Symmetry codes: i: 1 - x, -y, -z

**Fig. 2.**
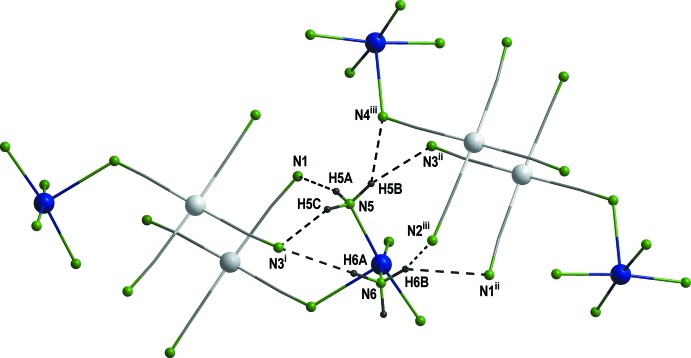
View on the intermolecular hydrogen bonds (dashed lines) of 1. Symmetry codes: i: 1 - x, 0.5 + y, 0.5 - z; ii: 0.5 + x, 0.5 - y, -0.5 - z; iii: 1.5 - x, 1- y, -0.5 + z

## supplementary crystallographic information

### Comment

The compound [Cu(NH_3_)_4_-(µ_2_-CN)—Pd(CN)_3_] (1) is formed by [Pd(CN)_4_]^2-^ and [Cu(NH_3_)_4_]^2+^ units held together by a bridging CN group (Fig. 1). In the solid state, the [Pd(CN)_4_]^2-^ anion is located on a mirror plane, while from cation only copper(II) atom lies on it and the [Cu(NH_3_)_4_]^2+^ group is bisected by this crystallographic symmetry plane. All of the atoms except those of the NH_3_ ligands reside on special positions. The Pd(II) atom is coordinated by four cyanido ligands and the Cu(II) atom is coordinated by four NH_3_ groups and one CN bridging ligand forming a square pyramid around copper(II) (τ = 0). The distance Cu—N4, from copper to the nitrogen atom of the bridging cyanido ligand, has a value of 2.385 (4) Å and is larger than bonds between copper(II) and nitrogen atoms from ammin ligand (2.016 (3) and 2.024 (3) Å). For example this distance can be compared to the Cu—N(apical) distance of 2.394 (7) Å found in [Cu(NH_3_)_4_Pt(CN)_4_] (Escorihuela *et al.*, 2001). This bond is perpendicular to the CuN_4_ plane, as expected for square-based pyramidal coordination environment. The Pd—C(bridge) distance (1.985 (5) Å) is smaller than Pd—C(terminal) distances, but the difference is within experimental error (1.992 (5)–2.005 (5) Å). The molecules are packed in such a way that the [Pd(CN)_4_] groups are stacked, with the Pd atoms forming nearly linear chain with a Pd···Pd distance of 3.654 (1) Å and a Pd···Pd···Pd angle of 163.97 (1)°. It is noteworthy that Cu—N≡C angle, which has values of 122.5 (4)°, should be collinear with the triple bond, but based on our previous study of the similar compounds observed angle is not uncommon in such compounds. For example, the Cu—N≡ C angle in [Cu(en)_2_Ni(CN)_4_]_n_ is 123.1 (1)°, with a Cu—N distance of 2.492 (3) Å (Seitz *et al.*, 2001) and [Cu(dmen)_2_Pd(CN)_4_]_n_ has Cu—N≡C angle of 138.0 (1)°, with corresponding Cu—N distance of 2.537 (1) Å (Kuchár *et al.*, 2004). An explanation for the bended structure of [Cu(NH_3_)_4_-(µ_2_-CN)—Pd(CN)_3_] is revealed by the extensive network of the intra- and intermolecular interactions in which the complex participates (see Table 1, Fig. 2). Each nitrogen atom of the terminal cyanide groups is involved in at least two crystallographically unique interactions, one with an N—H···N angle greater than 150° and the other corresponding to the bifurcated hydrogen. Moreover, since the CN groups reside on mirror planes and the donor atoms do not, the total number of interactions in which the CN ligand acts as an acceptor is at least 4 for each nonbridging cyanide. It is obvious that this three-dimensional electrostatic net plays an important role in establishing the deformation observed in the Cu—N≡C angle.

### Experimental

To 10 cm^3^ of 0.1 *M* CuSO_4_ solution (1 mmol) under continuous stirring 10 cm^3^ of 0.1 *M* butane-1,4-diamine solution (1 mmol) was added, followed by addition of 10 ml of 0.1 *M* K_2_[Pd(CN)_4_] solution (1 mmol). Created insoluble precipitate was dissolved by addition of ammonium hydroxide in excess amount. The formed clear blue solution was left for crystallization at room temperature. Single crystals of 1, in the form of blue needles suitable for X-ray studies, appeared after one day. As the crystals lost transparency and color upon being removed from solution in short time, the crystal used for X-ray was removed from solution without washing and drying and put on diffractometer. Attempt for direct preparation (without butane-1,4-diamine) does not produce any suitable product for X-ray. Elemental analysis was not performed because of the behavior of the product in the absence of ambient ammonia. The IR-spectrum in form of KBr pellets was recorded on a Avatar 330 F T—IR spectrophotometer (Thermo Nicolet) and following absorption bands were observed (4000–400 cm^-1^, s = strong, m = medium, w = weak, v = very): ν(NH): 3360(*vs*), 3273(*vs*), 3182(*s*); ν(CN): 2179(*s*), 2156(*m*), 2148(*s*), 2133(*vs*); δ(NH_2_): 1618(*m*); δ(NH_3_): 1280(*m*), 1263(*s*); δ(NH_2_): 696(*s*); δ(Pd—CN): 402(*s*).

### Refinement

The structure was solved by direct method. Anisotropic thermal parameters were refined for all non-H atoms. All H atoms positions were calculated using the appropriate riding model with isotropic temperature factors being 1.5 times larger then temperature factors of their parent nitrogen atoms and with N—H = 0.910 Å. Geometrical analysis was performed using *SHELXL97* (Sheldrick, 2008).

### Crystal data

**Table:**

[CuPd(CN)_4_(NH_3_)_4_]	*F*(000) = 668
*M**_r_* = 342.17	*D*_x_ = 2.081 Mg m^−^^3^
Orthorhombic, *P**n**m**a*	Mo *K*α radiation, λ = 0.71073 Å
Hall symbol: -P 2ac 2n	Cell parameters from 1245 reflections
*a* = 14.5204 (9) Å	θ = 2.4–26.7°
*b* = 7.2358 (5) Å	µ = 3.57 mm^−^^1^
*c* = 10.3955 (6) Å	*T* = 100 K
*V* = 1092.22 (12) Å^3^	Needle, blue
*Z* = 4	0.3 × 0.1 × 0.1 mm

### Data collection

**Table:**

Stoe IPDS-II diffractometer	1051 independent reflections
Radiation source: fine-focus sealed tube	958 reflections with *I* > 2σ(*I*)
Graphite monochromator	*R*_int_ = 0.032
Detector resolution: 150 pixels mm^-1^	θ_max_ = 25.0°, θ_min_ = 2.4°
ω scans	*h* = −16→17
Absorption correction: multi-scan (Blessing, 1995)	*k* = −8→8
*T*_min_ = 0.332, *T*_max_ = 0.646	*l* = −10→12
4036 measured reflections	

### Refinement

**Table:**

Refinement on *F*^2^	Primary atom site location: structure-invariant direct methods
Least-squares matrix: full	Secondary atom site location: difference Fourier map
*R*[*F*^2^ > 2σ(*F*^2^)] = 0.025	Hydrogen site location: inferred from neighbouring sites
*wR*(*F*^2^) = 0.061	H-atom parameters constrained
*S* = 1.00	*w* = 1/[σ^2^(*F*_o_^2^) + (0.0469*P*)^2^] where *P* = (*F*_o_^2^ + 2*F*_c_^2^)/3
1051 reflections	(Δ/σ)_max_ = 0.001
81 parameters	Δρ_max_ = 0.54 e Å^−^^3^
0 restraints	Δρ_min_ = −1.26 e Å^−^^3^

### Special details

**Table:**

Geometry. All e.s.d.'s (except the e.s.d. in the dihedral angle between two l.s. planes) are estimated using the full covariance matrix. The cell e.s.d.'s are taken into account individually in the estimation of e.s.d.'s in distances, angles and torsion angles; correlations between e.s.d.'s in cell parameters are only used when they are defined by crystal symmetry. An approximate (isotropic) treatment of cell e.s.d.'s is used for estimating e.s.d.'s involving l.s. planes.
Refinement. Refinement of *F*^2^ against ALL reflections. The weighted *R*-factor *wR* and goodness of fit *S* are based on *F*^2^, conventional *R*-factors *R* are based on *F*, with *F* set to zero for negative *F*^2^. The threshold expression of *F*^2^ > σ(*F*^2^) is used only for calculating *R*-factors(gt) *etc*. and is not relevant to the choice of reflections for refinement. *R*-factors based on *F*^2^ are statistically about twice as large as those based on *F*, and *R*- factors based on ALL data will be even larger.

### Fractional atomic coordinates and isotropic or equivalent isotropic displacement parameters (Å^2^)

**Table:**

	*x*	*y*	*z*	*U*_iso_*/*U*_eq_	
Pd1	0.48951 (2)	0.2500	0.01963 (3)	0.01617 (14)	
Cu1	0.80131 (4)	0.2500	−0.13557 (5)	0.01636 (17)	
N1	0.5331 (3)	0.2500	−0.2768 (4)	0.0219 (8)	
N2	0.4665 (3)	0.2500	0.3197 (4)	0.0245 (8)	
N3	0.2732 (3)	0.2500	−0.0018 (4)	0.0199 (8)	
N4	0.7043 (3)	0.2500	0.0495 (4)	0.0204 (8)	
C1	0.5119 (3)	0.2500	−0.1707 (5)	0.0204 (10)	
C2	0.4741 (3)	0.2500	0.2100 (5)	0.0198 (9)	
C3	0.3529 (3)	0.2500	0.0004 (4)	0.0189 (9)	
C4	0.6252 (3)	0.2500	0.0423 (4)	0.0193 (9)	
N5	0.73048 (18)	0.4477 (4)	−0.2315 (3)	0.0201 (5)	
H5A	0.6749	0.4019	−0.2563	0.030*	
H5B	0.7630	0.4831	−0.3022	0.030*	
H5C	0.7217	0.5469	−0.1791	0.030*	
N6	0.88190 (18)	0.4483 (3)	−0.0588 (3)	0.0201 (5)	
H6A	0.8470	0.5492	−0.0400	0.030*	
H6B	0.9265	0.4801	−0.1162	0.030*	
H6C	0.9086	0.4050	0.0145	0.030*	

### Atomic displacement parameters (Å^2^)

**Table:**

	*U*^11^	*U*^22^	*U*^33^	*U*^12^	*U*^13^	*U*^23^
Pd1	0.0149 (2)	0.01665 (19)	0.0170 (2)	0.000	−0.00032 (13)	0.000
Cu1	0.0168 (3)	0.0143 (2)	0.0180 (3)	0.000	−0.0003 (2)	0.000
N1	0.022 (2)	0.0249 (18)	0.019 (2)	0.000	−0.0015 (18)	0.000
N2	0.026 (2)	0.0241 (18)	0.023 (2)	0.000	0.0010 (19)	0.000
N3	0.020 (2)	0.0185 (17)	0.021 (2)	0.000	−0.0030 (17)	0.000
N4	0.018 (2)	0.0222 (17)	0.021 (2)	0.000	0.0015 (16)	0.000
C1	0.014 (2)	0.017 (2)	0.029 (3)	0.000	−0.005 (2)	0.000
C2	0.015 (2)	0.0184 (19)	0.026 (3)	0.000	0.000 (2)	0.000
C3	0.024 (3)	0.0148 (18)	0.018 (2)	0.000	−0.002 (2)	0.000
C4	0.028 (3)	0.0125 (18)	0.017 (2)	0.000	−0.001 (2)	0.000
N5	0.0212 (13)	0.0188 (11)	0.0201 (13)	−0.0018 (11)	0.0011 (11)	−0.0012 (10)
N6	0.0178 (13)	0.0198 (11)	0.0227 (13)	0.0003 (10)	0.0023 (11)	0.0009 (11)

### Geometric parameters (Å, º)

**Table:**

Pd1—C4	1.985 (5)	N2—C2	1.145 (6)
Pd1—C2	1.992 (5)	N3—C3	1.157 (6)
Pd1—C3	1.994 (5)	N4—C4	1.150 (6)
Pd1—C1	2.005 (5)	N5—H5A	0.9100
Cu1—N6	2.016 (3)	N5—H5B	0.9100
Cu1—N6^i^	2.016 (3)	N5—H5C	0.9100
Cu1—N5^i^	2.024 (3)	N6—H6A	0.9100
Cu1—N5	2.024 (3)	N6—H6B	0.9100
Cu1—N4	2.385 (4)	N6—H6C	0.9100
N1—C1	1.146 (7)		
			
C4—Pd1—C2	89.63 (18)	N1—C1—Pd1	173.7 (4)
C4—Pd1—C3	178.93 (18)	N2—C2—Pd1	179.1 (4)
C2—Pd1—C3	89.31 (17)	N3—C3—Pd1	175.4 (4)
C4—Pd1—C1	87.49 (17)	N4—C4—Pd1	176.9 (4)
C2—Pd1—C1	177.11 (17)	Cu1—N5—H5A	109.5
C3—Pd1—C1	93.58 (17)	Cu1—N5—H5B	109.5
N6—Cu1—N6^i^	90.73 (15)	H5A—N5—H5B	109.5
N6—Cu1—N5^i^	173.08 (11)	Cu1—N5—H5C	109.5
N6^i^—Cu1—N5^i^	89.26 (11)	H5A—N5—H5C	109.5
N6—Cu1—N5	89.26 (11)	H5B—N5—H5C	109.5
N6^i^—Cu1—N5	173.08 (11)	Cu1—N6—H6A	109.5
N5^i^—Cu1—N5	89.91 (15)	Cu1—N6—H6B	109.5
N6—Cu1—N4	91.35 (10)	H6A—N6—H6B	109.5
N6^i^—Cu1—N4	91.35 (10)	Cu1—N6—H6C	109.5
N5^i^—Cu1—N4	95.57 (10)	H6A—N6—H6C	109.5
N5—Cu1—N4	95.57 (10)	H6B—N6—H6C	109.5
C4—N4—Cu1	122.5 (4)		
			
N6—Cu1—N4—C4	134.62 (7)	N5^i^—Cu1—N4—C4	−45.23 (8)
N6^i^—Cu1—N4—C4	−134.62 (7)	N5—Cu1—N4—C4	45.23 (8)

Symmetry code: (i) *x*, −*y*+1/2, *z*.

### Hydrogen-bond geometry (Å, º)

**Table:**

*D*—H···*A*	*D*—H	H···*A*	*D*···*A*	*D*—H···*A*
N5—H5*A*···N1	0.91	2.34	3.237 (4)	167
N5—H5*C*···N3^ii^	0.91	2.39	3.267 (4)	162
N5—H5*B*···N3^iii^	0.91	2.65	3.180 (4)	118
N5—H5*B*···N4^iv^	0.91	2.52	3.297 (4)	144
N6—H6*B*···N1^iii^	0.91	2.53	3.131 (4)	124
N6—H6*B*···N2^iv^	0.91	2.58	3.348 (4)	142
N6—H6*A*···N3^ii^	0.91	2.31	3.199 (4)	165

Symmetry codes: (ii) −*x*+1, −*y*+1, −*z*; (iii) *x*+1/2, *y*, −*z*−1/2; (iv) −*x*+3/2, −*y*+1, *z*−1/2.
